# Experimental and meta-analytic evidence that source variability of misinformation does not increase eyewitness suggestibility independently of repetition of misinformation

**DOI:** 10.3389/fpsyg.2023.1201674

**Published:** 2023-08-24

**Authors:** Rachel O’Donnell, Jason C. K. Chan, Jeffrey L. Foster, Maryanne Garry

**Affiliations:** ^1^Memory, Law, and Education Laboratory, Psychology Department, Iowa State University, Ames, IA, United States; ^2^Department of Security Studies and Criminology, Macquarie University, Sydney, NSW, Australia; ^3^School of Psychology, The University of Waikato, Hamilton, New Zealand

**Keywords:** eyewitness memory, misinformation, repetition, source variability, eyewitness suggestibility, misinformation effect

## Abstract

Considerable evidence has shown that repeating the same misinformation increases its influence (i.e., repetition effects). However, very little research has examined whether having multiple witnesses present misinformation relative to one witness (i.e., source variability) increases the influence of misinformation. In two experiments, we orthogonally manipulated repetition and source variability. Experiment 1 used written interview transcripts to deliver misinformation and showed that repetition increased eyewitness suggestibility, but source variability did not. In Experiment 2, we increased source saliency by delivering the misinformation to participants via videos instead of written interviews, such that each witness was visibly and audibly distinct. Despite this stronger manipulation, there was no effect of source variability in Experiment 2. In addition, we reported a meta-analysis (*k* = 19) for the repeated misinformation effect and a small-scale meta-analysis (*k* = 8) for the source variability effect. Results from these meta-analyses were consistent with the results of our individual experiments. Altogether, our results suggest that participants respond based on retrieval fluency rather than source-specifying information.

## Introduction

Research on the misinformation effect (when exposure to misleading information harms memory performance) has contributed greatly to the understanding of the fallibility of human memory. Despite its replicability, most of the research in the misinformation literature has used variants of the same three-phase paradigm, which consists of (i) participants witnessing an event, (ii) being introduced to misinformation, and (iii) taking a memory test. Most studies using this paradigm provided misinformation to participants using a single source (e.g., participants might be introduced to misinformation by reading a narrative purportedly written by a professor; Zaragoza et al., 2007; Berkowitz and Loftus, 2018). But crimes are often witnessed by multiple people, so eyewitnesses may be introduced to misinformation multiple times and/or through multiple sources (Clark and Wells, 2008; Skagerberg and Wright, 2008). For example, co-witnesses to a crime might discuss the details of the event with each other, during which incorrect information might be introduced, and the misinformation might be repeated by the same or other co-witnesses. Given the important role that eyewitnesses play in criminal investigations, it is crucial to understand how an eyewitness’ memory may be influenced by receiving the same piece of misinformation multiple times and through more than one source (e.g., from multiple people). Although some reports have shown that repeated exposure to misinformation can exacerbate its influence (Mitchell and Zaragoza, 1996; Walther et al., 2002; Ecker et al., 2011; Bright-Paul and Jarrold, 2012; Schwarz et al., 2016; Ecker et al., 2020), very little research has independently examined the effects of repetition and source variability on eyewitness suggestibility (Mitchell and Zaragoza, 1996; Foster et al., 2012).

The purpose of the current study was to examine how source variability and repetition of misinformation influence eyewitness suggestibility. Source variability was defined as the number of people who delivered the misinformation, and repetition was defined as the number of presentations of the same misinformation. Below, we first review the literature regarding the effect of repetition of misinformation on suggestibility, we then review the literature on memory conformity that pertains to source variability, and we lastly review the previous studies that have investigated both repetition and source variability of misinformation (Mitchell and Zaragoza, 1996; Foster et al., 2012).

### Repeated exposure to misinformation

In general, repeated exposure to misinformation increases the misinformation effect (Mitchell and Zaragoza, 1996; Zaragoza et al., 2007; Ecker et al., 2011; Bright-Paul and Jarrold, 2012; Foster et al., 2012). This repetition effect has been observed across different participant populations (see Mitchell and Zaragoza, 1996; Bright-Paul and Jarrold, 2012) and is thought to occur as a result of increased processing fluency or increased belief in the truthfulness of the misinformation (see Arkes et al., 1991; Hassan and Barber, 2021).

The illusory truth effect (Hasher et al., 1977; Dechêne et al., 2010) suggests that repeating misinformation might increase its believability. In the illusory truth paradigm, participants are asked to rate a series of plausible statements for truthfulness (“Lithium is the lightest of all metals”). The typical finding is that repetition increases ratings of truth. There are several predominant explanations for the illusory truth effect, but the source dissociation hypothesis and the processing fluency hypothesis are most relevant to this study. The first hypothesis proposes that successive repetitions increase the processing fluency of an item, and because truth and fluency are highly correlated, people tend to use fluency as a marker for truthfulness (Arkes et al., 1991; Hassan and Barber, 2021). The second hypothesis proposes that repetition increases a statement’s credibility because participants mistakenly attribute a prior presentation of the statement to an independent, outside source (Arkes et al., 1991; Roggeveen and Johar, 2002). Both the fluency and source dissociation hypotheses have received empirical support (Begg et al., 1992; Roggeveen and Johar, 2002; Henderson et al., 2021), and the mechanisms underlying each should apply regardless of whether participants are judging the truthfulness of correct statements or misinformation. Together, existing data suggest that repetition of (mis)information should increase eyewitness suggestibility.

### Memory conformity, credibility, and source variability

In contrast to the voluminous literature on repetition effects (Mitchell and Zaragoza, 1996; Zaragoza et al., 2007; Ecker et al., 2011; Bright-Paul and Jarrold, 2012; Foster et al., 2012), far less research has investigated whether source variability of misinformation might influence eyewitness suggestibility (Mitchell and Zaragoza, 1996; Foster et al., 2012), but data in the memory conformity and credibility literatures can provide a basis for predictions about the effects of source variability. Memory conformity studies, unlike most misinformation studies (which are typically carried out in solitary circumstances), were originally intended to investigate how participants conform to responses made by others (social influences). In some memory conformity studies, participants receive misinformation from a confederate posing as a co-witness, and participants often mistakenly report that misinformation on a later memory test (Reysen, 2007; Goodwin et al., 2013; Thorley, 2013). Memory conformity is often studied in the context of a single co-witness, but some research has shown that participants exhibited greater conformity when misinformation was provided by two or more co-witnesses (Ost et al., 2008; Jack et al., 2014). Similarly, when the same misinformation was delivered by multiple witnesses, it was judged more convincing than when it was provided by one witness (Lindsay et al., 1986). Extrapolating from these findings, presenting misinformation from multiple sources might increase eyewitness suggestibility.

A serious problem with the above-cited studies and many others in the memory conformity literature (Walther et al., 2002; Vrij et al., 2005; Mojtahedi et al., 2018) is that they have all confounded source variability with repetition, such that when misinformation was delivered by multiple people (increased sources), it was also repeated in each successive presentation (increased repetition). These studies, therefore, do not offer insight regarding whether the effect of group size occurs because of repetition or source variability.

Relatedly, much of the work in the credibility and misinformation effect literature has shown that eyewitnesses are more susceptible to misinformation when it is presented by a more credible source than a less credible one (Dodd and Bradshaw, 1980; Smith and Ellsworth, 1987). Of particular relevance is a study conducted by Park et al. (2017). In this study, participants read fictitious crime vignettes and then made punitive judgments for the suspects and provided confidence for these judgments. Participants were then given a chance to reconsider their judgments after being provided with the average decision of other mock jurors. Importantly, Park et al. manipulated the group size of the jury and found that participants were more likely to yield to the judgment of a putatively larger group than a smaller group. This finding suggests that participants might have regarded a decision made with more sources as one with greater consensus and credibility. Taken together, the results from the aforementioned literatures (i.e., illusory truth effect, memory conformity, group size, and credibility) suggest that when multiple witnesses provide misinformation, participants might be particularly susceptible to misinformation because of an increased perception of consensus.

### Studies that investigated repetition and source variability independently

To our knowledge, only one study to date has examined the effects of both source variability and repetition of misinformation on eyewitness memory (Foster et al., 2012). In Foster et al.’s study, participants watched a short video (~ 6 min) in which an electrician stole several items from a client’s house. Following a brief filler task, participants read three reports labeled as the transcript of a police interview, a written police interview, and the transcript of a follow-up interview. Participants were informed that the reports had been created by interviewing other participants in a previous experiment. To manipulate source information, each interview transcript was labeled with a witness identifier. In the one-witness (*1W*) condition, the same identifier (e.g., 9) appeared on all three transcripts; in the three-witness (*3W*) condition, different identifiers (e.g., 5, 9, 16) appeared on each transcript. Participants in the repeated-misinformation (*3X*) condition read three misleading transcripts, in which every piece of misinformation was presented once in each transcript, for a total of three presentations per misinformation. Participants in the nonrepeated-misinformation (*1X*) condition read one misleading transcript and two control transcripts, with the misleading transcript presented either first or last. Within the transcripts, each critical item (e.g., a black or blue cap, depending on the video version) was either misleading (a blue cap was *incorrectly* described as black and vice versa) or neutral (mentioning the cap without describing its color). In summary, misinformation was presented in one of four ways –one exposure via a single source (1X-1W), one exposure via three sources (1X-3W), three exposures via a single source (3X-1W), or three exposures via three sources (3X-3W). After reading the three transcripts, participants took a two-alternative forced choice (2AFC) recognition test (in which participants must choose one response option), with the correct answer and the misinformation serving as the response options. Foster and colleagues found that repetition, but not the number of sources of misinformation, reduced eyewitness memory accuracy.

Mitchell and Zaragoza (1996) also investigated a similar question, but their study did not specifically examine source variability. Here, participants viewed a short police training film and then answered 12 questions, with misinformation embedded in statements before some of the questions. Participants were presented with each set of misinformation zero, one, and three times, with each presentation occurring in a different modality (i.e., via printed paper, via audiotape, *and* via videotape) or the same modality (i.e., via printed paper, via audiotape, *or* via videotape). Finally, participants took a source memory test. Like Foster et al. (2012), Mitchell and Zaragoza found that repeated exposure to the same suggestions increased source misattributions relative to a single exposure. However, unlike Foster et al. when the misinformation was presented three times, participants in the mixed modality condition (which arguably produced more varied sources) made significantly more misattributions than those in the single modality condition. This finding demonstrated that a context manipulation – enacted via presentation modality – increased participants’ suggestibility independently of repetition.

It is not clear what contributed to the discrepancies regarding the effects of misinformation presentation context between Foster et al. (2012) and Mitchell and Zaragoza (1996), but one possibility is that Foster et al. varied context via misinformation sources (such as the number of witnesses) whereas Mitchell and Zaragoza varied context via modality. The latter method might have made the context manipulation more salient to participants, thereby enhancing its effects. In particular, the source variability manipulation in Foster et al. – by marking the cover sheet of each interview transcript with a different numeric identifier – might have been too subtle. Specifically, it is possible that participants might not have paid attention to the witness identifier when they read the interview transcripts. If this were the case, participants in the three-witness condition would not remember that they had read transcripts allegedly produced by three different people, thereby rendering the source variability manipulation ineffective. Even if participants had attended the cover page, the written reports did not differ in any perceptually obvious ways, so it might be difficult for participants to distinguish the sources. In two preregistered experiments, we sought to further investigate the effects of repetition and source variability on eyewitness suggestibility. After attempting to conceptually replicate Foster et al.’s study in our Experiment 1, we aimed to boost the salience of our source manipulation in an ecologically realistic manner in Experiment 2.

## The current experiments

The goal of the present study was to examine the extent to which source variability and repetition of misinformation influence eyewitness suggestibility. Both experiments were preregistered on the Open Science Framework (OSF), and our experimental materials and data are available at https://osf.io/9zpfk/?view_only=f95ed70720c742d48296fa3b92891ed7. In addition to the two experiments, we also conducted two non-preregistered meta-analyses to further examine the influence of our independent variables (repetition and source variability) on the misinformation effect. We report the results of these meta-analyses at the end of our results section before the General Discussion. To briefly preview, only the current studies and Foster et al. have independently examined the influence of source variability on the misinformation effect, so the source variability meta-analysis included data from only those studies.

Experiment 1 was an attempted conceptual replication of Foster et al.’s Experiment 1^1^ Foster et al. (2012) using novel materials. We hypothesized that Experiment 1 would replicate the results of Foster et al., such that the repetition manipulation (three presentations of misinformation relative to one) would decrease participants’ response accuracy, but the source variability manipulation (three sources of misinformation relative to one) would not.

In Experiment 2, we attempted to create a more salient source variability manipulation. To this end, we presented the interviews as videos, rather than written transcripts, with three different actors. Some research has suggested that misinformation delivered “directly” – by providing social cues like appearance and mannerisms – creates a stronger misinformation effect than misinformation delivered “indirectly” (one that does not provide social cues; Gabbert et al., 2004; Blank et al., 2013). Although the delivery in this case was not done in person, the videos provided rich source-specifying information about each witness (e.g., they looked different, sounded different, and had different mannerisms), and participants could draw on these distinctive, source-specifying details to distinguish the sources. Therefore, we hypothesized that participants would be more suggestible when they received misinformation from multiple people (via videos) relative to one person, especially when the misinformation was repeated.

**Figure 1:**
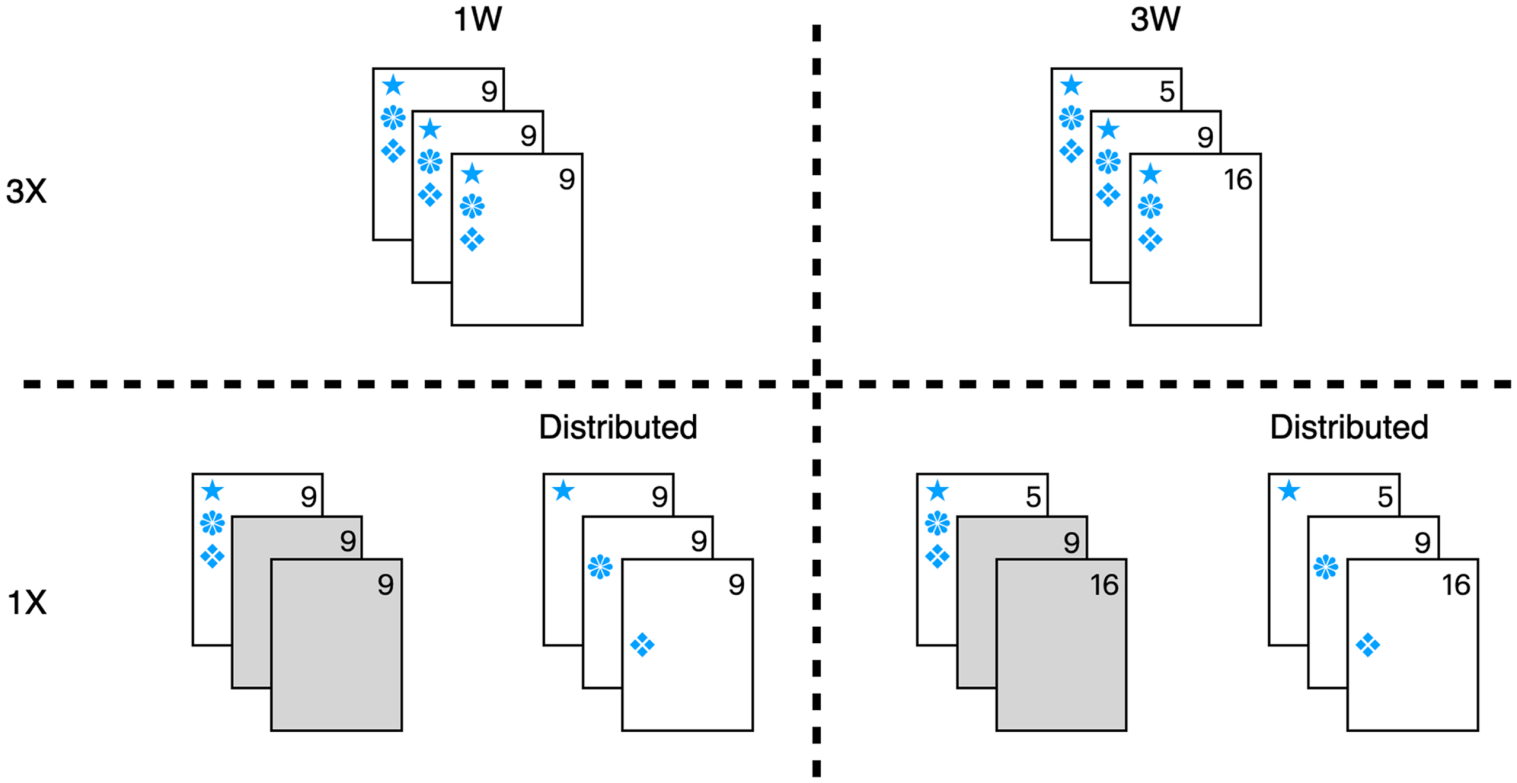
Illustration of critical items contained in each interview type and condition. Control interviews, with no misleading items, are indicated in gray. Each symbol represents a different piece of misinformation. In the 3X condition, each piece of misinformation appeared in all three interviews. In the 1X condition, all misinformation appeared only once and in a single interview. In the 1X-Distributed condition, all misinformation appeared only once, but the misinformation was distributed across three interviews.

The design and procedure of both experiments were modeled after Foster et al. (2012). In our Experiment 1, all participants viewed a video and then read three interview transcripts, and misinformation was presented once (1X) or three times (3X), either from one witness (1W) or from three witnesses (3W). In the 1X condition, the misinformation was presented in only one interview, and this interview was presented either first or last. In addition to replicating these conditions from Foster et al. we also created an *additional* 1X condition that distributed the misinformation throughout all three interviews and termed these the *1X distributed* conditions so that every interview presented misinformation, regardless of whether one or three witnesses provided misinformation. Therefore, each experiment had six conditions–(i) 1W-3X, (ii) 3W-3X, (iii) 1W-1X, (iv) 3W-1X, (v) 1W-1X distributed, and (vi) 3W-1X distributed (see Table 1). Figure 1 depicts distribution of the critical items visually.

**Table 1:** Distribution of misleading claims in each condition.

Condition	Distribution of misleading claims
1W-3X	One witness made the same six misleading claims in each of the three interviews
3W-3X	Three different witnesses made the same six misleading claims in each of the three interviews
1W-1X	One witness made six misleading claims in only one interview
3W-1X	Three witnesses, one of whom made six misleading claims in only one interview
1W-1X Distributed	One witness made two misleading claims in each of the three interviews
3W-1X Distributed	Three witnesses each made two misleading claims in each of the three interviews

Underlines indicate conditions that were in Foster et al. (2012).

**Figure 2:**
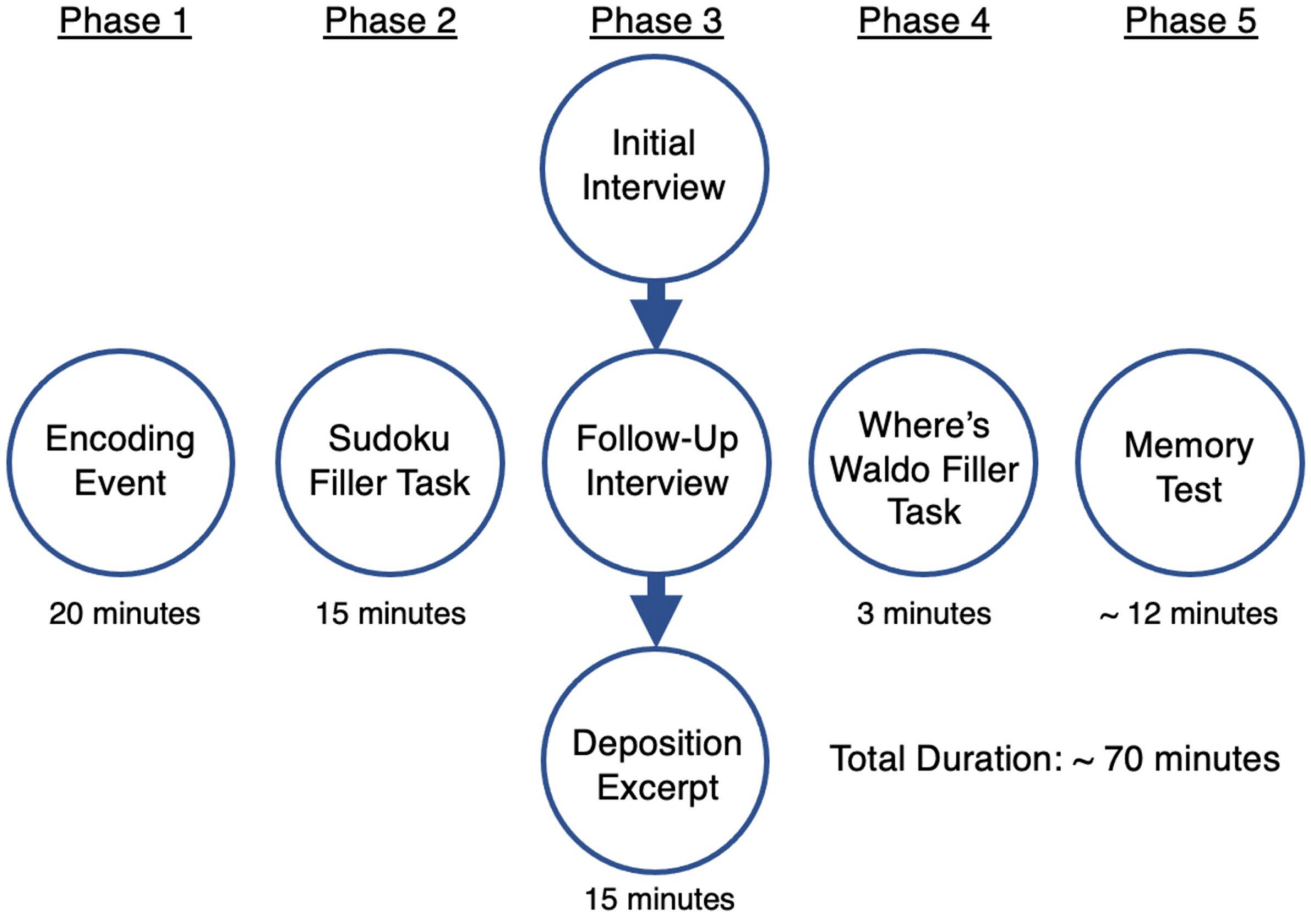
Illustration of procedure for experiments 1 and 2. The figure shows the estimated completion time for Experiment 1. Experiment 2’s estimated completion time was ~90 min due to the length of the interview videos.

See Figure 2 for an illustration of the procedure. In Experiment 1, participants first watched an encoding event that depicted a robbery and then read three interview transcripts. The interviews were formatted as an initial interview, a follow-up interview, and a deposition excerpt. Modeling after Foster et al. (2012), each interview had a cover page that described when the interview occurred along with a large, handwritten code that indicated who provided the interview (e.g., 9 for the 1W condition, and 5, 9, and 16 for the 3W condition). Finally, participants took a 2AFC recognition test. The design and procedure of Experiment 2 were the same as Experiment 1, except that each interview was presented as a video featuring different actresses to ensure that the source differences were obvious to participants.

## Experiment 1

### Participants

A power analysis was conducted to determine sample size. The estimated effect size of misinformation repetition was *d* = 0.64 based on data from Foster et al. (2012). Because Foster et al. did not report a significant effect of source variability on suggestibility, we chose the smallest effect size of interest (*d* = 0.25). We conducted a power analysis for comparison of a main effect, with a Cohen’s *d* of 0.25 and power of 0.50 (one-tailed, α = 0.05). The minimum sample size per group was 88 (or 44 per condition), so we aimed to collect data from 264 participants. Note that this sample size provided 0.99 power to detect the repetition main effect of *d* = 0.64 in a two-tailed test at alpha = 0.05. Participants were undergraduate students from Iowa State University who participated for course credit.

All data were collected online via Qualtrics due to COVID-19. A total of 310 participants completed Experiment 1,^2^ but data from 43 participants were excluded from analysis (see Table 2 for exclusions and demographic information). The exclusion criteria were preregistered before data collection. Most participants were excluded based on their responses to the survey at the conclusion of the experiment, in which participants self-reported their proficiency in English, if they took the experiment seriously, edited the video in any way, had seen the encoding event before, or experienced any technical issues during the experiment. Other participants were excluded based on their responses to attention checks (Captcha, participation in the filler activities) or survey metadata (duration, devices). The final sample included 267 participants.

**Table 2:** Number of excluded participants, participants per condition, and demographic information in Experiments 1 and 2.

	E1	E2
*Reason for exclusion*		
Completed experiment in more than one session	16	9
Did not complete filler tasks (Sudoku, Where’s Waldo)	8	8
Edited, paused, or rewatched encoding event or interviews	8	26
Self-reported being not serious or not alert during experiment	5	19
Previously seen encoding event (within last six months)	3	8
Self-reported taking notes during encoding event or interviews	2	3
Duration of experiment exceeded 2 h	1	10
Experienced technical issues (i.e., W-Fi connection)	–	6
Self-reported low English language proficiency	–	4
Completed the study on a mobile device	–	3
Did not agree to the conditions on the consent form	–	3
*Participants retained per condition*		
1W-3X	44	45
3W-3X	45	45
1W-1X	44	45
3W-1X	46	45
1W-1X Distributed	44	44
3W-1X Distributed	44	44
*Ethnicity*		
White or Caucasian	82%	79%
Hispanic or Latinx	5%	6%
East Asian	4%	3%
Black or African American	2%	3%
South/Southeast Asian	2%	3%
West Asian/Middle Eastern	2%	2%
Native Hawaiian or Other Pacific Islander	1%	1%
Other	1%	2%
Chose not to respond	< 1%	< 1%
*Gender*		
Female	57%	57%
Male	42%	42%
Other	< 1%	1%

### Design

In addition to the six between-subjects conditions (illustrated in Table 1), item type (misleading or neutral) was manipulated within-subjects. For the 1X conditions, all of the critical items were presented in either the first or last interview (loading: first, last).

### Materials and procedure

The experiment contained five phases (see Figure 2). In Phase 1, participants watched a 20-min excerpt of an episode from the Canadian television show *Flashpoint* (season 1 episode 5). In the video, a former security guard named George attempted to rob the bank where he was employed. The police were called to the scene, and Sergeant Gregory Parker negotiated with George, but George threatened to kill the hostages. The video ended after the police rescued all the hostages except for the bank manager Ruth.

In Phase 2, participants completed a 15-min filler task in which they worked on two Sudoku puzzles (see OSF page for materials), and participants were automatically advanced to Phase 3 after the task.

**Figure 3:**
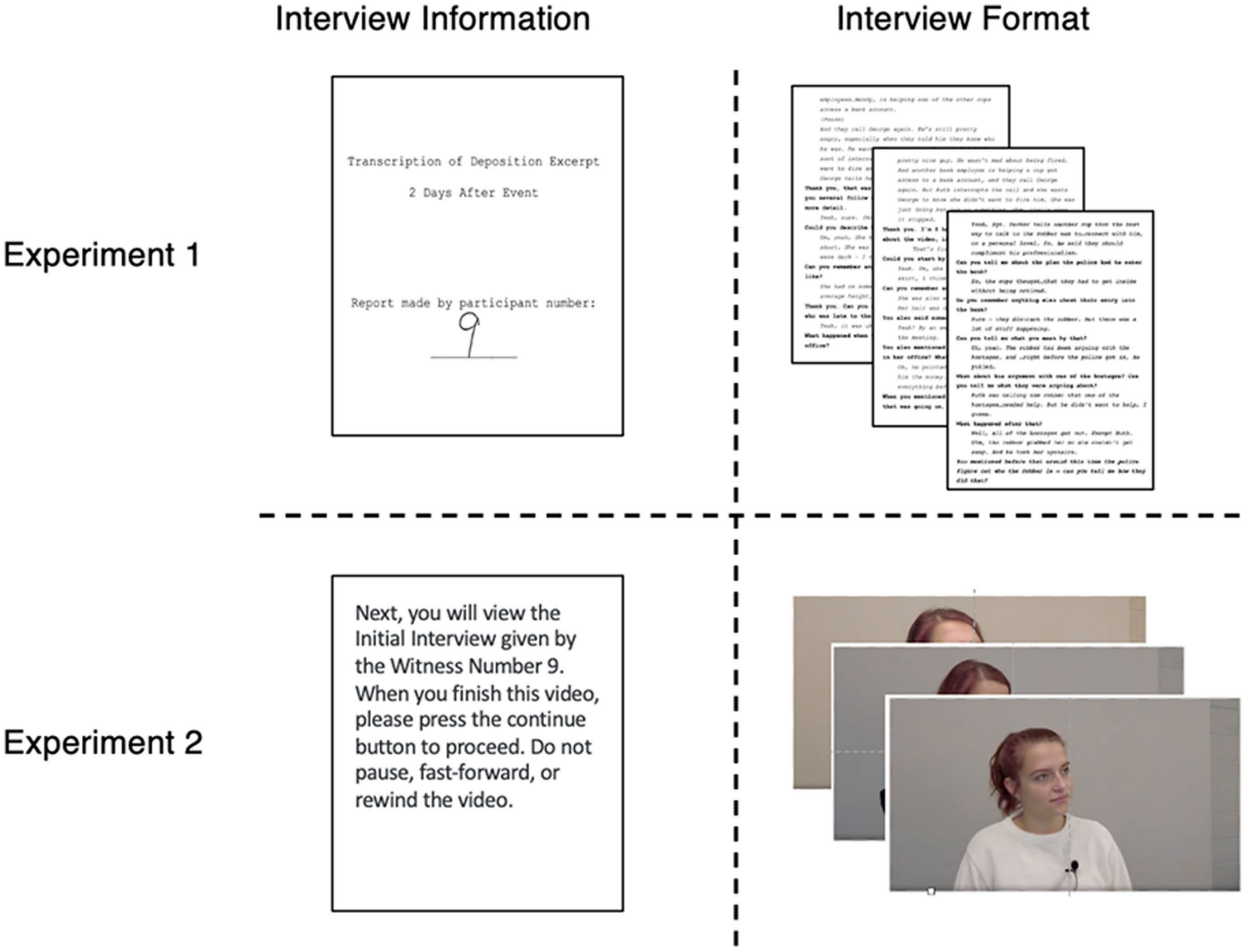
Illustration of interview information and format for Experiments 1 and 2.

In Phase 3, participants read three interviews. They were informed that a highly trained experimenter had conducted the interviews with participants from a previous experiment. The interviews were presented as an initial interview, a follow-up interview, and a simulated deposition excerpt. The interview transcripts were presented sequentially without breaks. Reading of the interviews was self-paced, but participants were required to spend at least 3 min on each interview. Each interview had a cover page with the handwritten eyewitness identifier (see the top panel of Figure 3) and the day the interview was conducted (“Day of Event,” “Day After Event,” and “Two Days After Event”).

In the 3X condition, all 12 critical items appeared in each of the three interview transcripts. In the 1X condition, all critical items appeared in only the first or last interview. In the 1X distributed condition, the critical items were spread across the three interviews, with each interview presenting four different items (see Figure 1). For example, the name of the bank, “City Central,” was a critical item. The *misleading* version of this critical detail named the bank “City Towers,” whereas the *neutral* version omitted the bank’s name. The assignment of each critical detail as misleading or neutral was counterbalanced across participants. Each interview also contained 12 filler items that were presented either three times in the 3X condition or once in the 1X conditions. These filler items were included so that the memory test queried both items that were presented correctly and incorrectly in the interviews rather than querying only omitted (neutral) or incorrect (misled) items.

Pilot testing was conducted to ensure the critical items produced a significant misinformation effect. Here, participants completed a condensed version of Experiment 1 without the main manipulations of repetition and source variability (*N* = 73 total participants in two rounds of pilot testing). The single interview contained 14 critical items (misleading or neutral) and 14 filler items. After an item analysis was conducted, two critical items were removed (low misinformation effect, < 5%), and two filler items were removed (ceiling performance, > 97%). The remaining 12 items produced an average misinformation effect of 21% (*d* = 1.12). Additional details about pilot testing can be found on the OSF project page.

Phase 4 included a 3-min “Where’s Waldo” filler task. Participants searched for the cartoon character Waldo (see OSF page for materials) in four pictures.

In Phase 5, participants completed a 24-question 2AFC recognition test (see OSF page for all questions). Twelve questions queried the 12 critical items (where the answer choices were either the correct answer or the misinformation), and 12 questions queried the filler items (where the answer choices were either the correct answer or an incorrect foil). Four filler questions with the highest accuracy (*M* = 0.89) in the pilot were always presented at the beginning of the test so that it would not be perceived as too difficult. The order of the remaining questions and answer choices (for all questions) was randomized. Participants also rated their confidence for each question on a scale from 1 to 5, with 1 meaning not at all confident and 5 meaning very confident. At the conclusion of the study, all participants completed a brief demographic questionnaire with manipulation check questions.^3^ Following completion of the questionnaire, all participants were debriefed.

### Results and discussion

We first report results of the same analyses as Foster et al. (2012) to determine whether we successfully replicated their recognition results (a 2x2x2 repeated measures ANOVA), and then report follow up *t*-tests to examine both the effect of repeating misinformation and multiple sources of misinformation on eyewitness accuracy. We report these analyses for the conditions that most closely replicated Foster et al. first, and then those that controlled for the distribution of misinformation. Finally, we conducted confidence-accuracy calibration analysis to further examine how item type (misled items vs. neutral items) influenced that relationship (this analysis was not conducted in Foster et al. and as such, is not the target of the replication).

We first reported the 3X vs. 1X comparison (the conditions most similar to Foster et al.) and then the 3X vs. 1X distributed comparison. An independent samples t-test showed that in the 1X condition, participants’ recognition accuracy did not differ significantly regardless of whether misinformation was presented in the first or third interview (all *t*s < 0.92, *p*s > 0.113), so the remaining analyses were collapsed across this variable. The selection rates for the filler items are displayed in Table 3.

**Table 3:** Recognition performance for filler items in Experiments 1 and 2 per condition.

	Accuracy
*Experiment 1*	
1W-3X	0.91 (0.12)
3W-3X	0.93 (0.11)
1W-1X	0.93 (0.13)
3W-1X	0.89 (0.13)
1W-1X Distributed	0.88 (0.14)
3W-1X Distributed	0.87 (0.13)
*Experiment 2*	
1W-3X	0.89 (0.12)
3W-3X	0.89 (0.14)
1W-1X	0.85 (0.18)
3W-1X	0.88 (0.14)
1W-1X Distributed	0.84 (0.15)
3W-1X Distributed	0.81 (0.16)

Standard deviations are represented in parentheses.

#### The effect of repetition and source variability on accuracy

##### Replication of Foster et al.’s conditions

**Figure 4:**
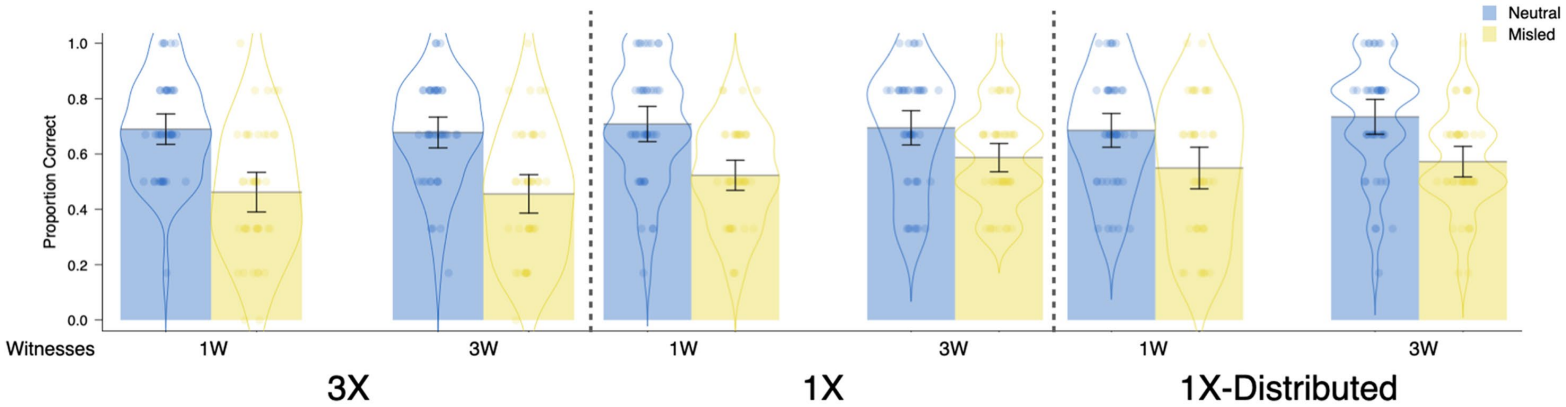
Proportion correct by item type in Experiment 1 as a function of repetition and sources. Each dot represents the data of an individual participant. Jitter was introduced to disperse the data points horizontally for visualization purposes. The violin element displays data density.

The most important findings are shown in Figure 4. Replicating the main findings from Foster et al. (2012), repetition of misinformation reduced participants’ recognition accuracy, but having three witnesses present misinformation did not affect recognition accuracy relative to one witness. The repetition results can be seen by comparing the left panel (3X) to the middle panel (1X) of Figure 4, and the source variability results can be seen by comparing the first (1W) to the second pair (3W) of bars within each panel.

The above impressions were realized in the results of a 2(repetition: 1X, 3X) × 2(source variability: 1W, 3W) × 2(item type: neutral, misled) repeated measures ANOVA with recognition accuracy (hit rate) as the dependent variable (Figure 4). This ANOVA revealed a main effect of item type, *F*(1, 175) = 78.90, *p* < 0.001, *d* = 0.66, which showed that participants’ accuracy was lower for misled items (*M* = 0.51, *SD* = 0.21) than neutral items (*M* = 0.69, *SD* = 0.20) – a misinformation effect. There was also a main effect of repetition, *F*(1, 175) = 6.89, *p* = 0.009, *d* = 0.20, a nonsignificant effect of source, *F*(1, 175) = 0.14, *p* = 0.713, *d* = 0.03, and a nonsignificant interaction between item type and repetition, *F*(1, 175) = 3.51, *p* = 0.063, η_p_^2^ = 0.02. All other effects were not significant, *F*s < 1.00, *p*s > 3.19.

Following Foster et al. (2012), we assessed the effects of repeating misinformation on eyewitness accuracy in separate t-tests. Critically, repetition of misinformation reduced recognition accuracy for the misled items (*M*_1X_ = 0.56, *M*_3X_ = 0.46), *t*(177) = 0.60, *p* = 0.002, *d* = 0.47, but not for the neutral items (*M*_1X_ = 0.70, *M*_3X_ = 0.68), *t*(177) = 0.60, *p* = 0.547, *d* = 0.09. In contrast to these results, having three witnesses deliver misinformation did not reduce recognition accuracy relative to having one witness deliver the same misinformation (accuracy for misled items: *M*_1W_ = 0.49, *M*_3W_ = 0.52, *t*[177] = 0.94, *p* = 0.350, *d* = −0.14; accuracy for neutral items: *M*_1W_ = 0.70, *M*_3W_ = 0.69, t[177] = 0.44, *p* = 0.662, *d* = 0.07).

##### Comparisons that controlled for the distribution of misinformation across interviews

Overall, the same conclusions as above were reached when we distributed the nonrepeated misinformation across all three interview transcripts (rather than presenting them in a single interview), such that repetition, but not source variability, increased the misinformation effect.

The following comparisons included the 1W-1X distributed, 1W-3X, 3W-1X distributed, and 3W-3X conditions. We again conducted a 2 × 2 × 2 ANOVA (Figure 4). There was again a main effect of item type, *F*(1, 173) = 82.29, *p* < 0.001, *d* = 0.68, which showed that participants were less accurate for misled items (*M* = 0.51, *SD* = 0.23) compared to neutral items (*M* = 0.70, *SD* = 0.19). There was also a main effect of repetition, *F*(1, 173) = 7.16, *p* = 0.008, *d* = 0.20, a nonsignificant effect of source, *F*(1, 173) = 0.31, *p* = 0.576, *d* = 0.04, and a nonsignificant interaction between item type and repetition, *F*(1, 173) = 3.38, *p* = 0.068, η_p_^2^ = 0.02. All other main effects and interactions were nonsignificant, *F*s < 0.89, *p*s > 0.348.

We again replicated the key results in Foster et al. (2012). Specifically, repetition of misinformation reduced participants’ accuracy for the misled items (*M*_1X_ = 0.56, *M*_3X_ = 0.46), *t*(175) = 3.03, *p* = 0.003, *d* = 0.46, but not for the neutral items (*M*_1X_ = 0.71, *M*_3X_ = 0.68), *t*(175) = 0.90, *p* = 0.369, *d* = 0.14. Moreover, the source variability manipulation did not influence participants’ accuracy for both misled items, *M*_1W_ = 0.51, *M*_3W_ = 0.51, *t*(175) = 0.22, *p* = 0.824, *d* = −0.03, and neutral items, *M*_1W_ = 0.69, *M*_3W_ = 0.71, *t*(175) = 0.62, *p* = 0.535, *d* = −0.09.

Across both comparisons in Experiment 1, we replicated the critical pattern of results found in Foster et al. (2012), such that repeating the same piece of misinformation three times reduced participants’ accuracy relative to presenting misinformation only once. In addition, we also found no effect of source variability – participants were no less accurate when they read interview transcripts marked as coming from three witnesses as opposed to a single witness.

#### Confidence-accuracy calibration by item type

To examine whether the relationship between confidence and accuracy varied by item type (for the critical items), we conducted a multilevel logistic regression analysis. We did not anticipate that either of the independent variables would affect the confidence-accuracy relationship, so we did not include them in the model. The multilevel model included data from all participants. Response accuracy (0 and 1) served as the dependent variable, and we regressed this variable on confidence, item type, and their interaction as fixed effects factors. The intercept was allowed to vary across participants as a random effects factor. We did not include any additional random effects factors because the model failed to converge when they were added.

**Figure 5:**
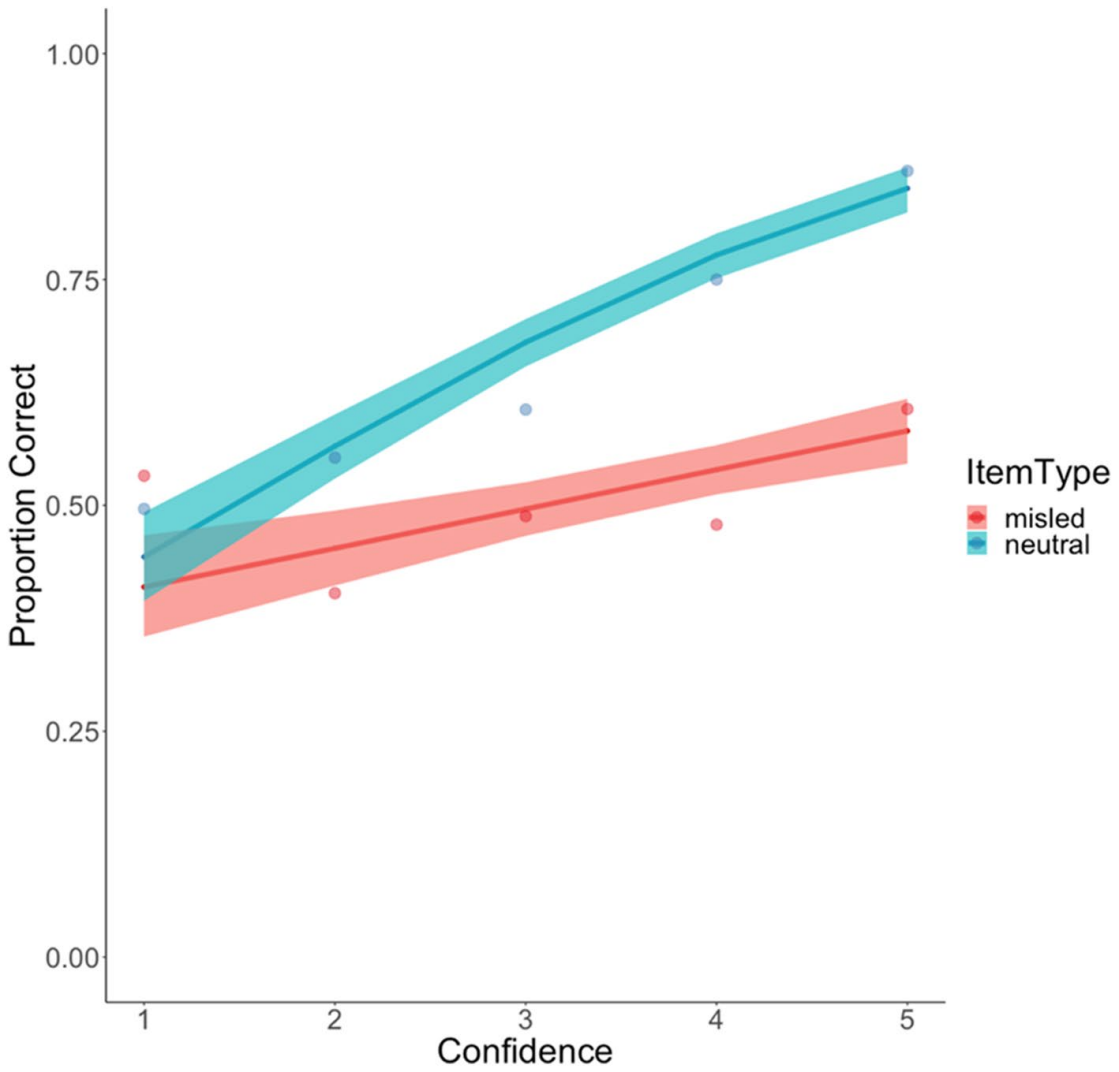
Confidence accuracy calibration as a function of item type in Experiment 1. Data points represent observed probabilities, and bands represent 0.95 CI for the fitted multilevel regression lines.

The most important result was a significant interaction between confidence and item type, *B* = 0.32, *SE* = 0.06, *z* = 5.767, *p* < 0.001, such that the confidence-accuracy relationship was much stronger for neutral items, *B* = 0.49, *SE* = 0.04, *z* = 12.27, *p* < 0.001, than for misled items, *B* = 0.17, *SE* = 0.04, *z* = 4.42, *p* < 0.001 – a pattern that is readily apparent in Figure 5. In fact, when viewing the observed data points, the confidence-accuracy relationship for the misled item was essentially flat, with participants performing at close to chance level across the entire confidence range. In contrast, as participants’ confidence rose, so did their recognition accuracy for the neutral items. Therefore, encountering misinformation severely undermined the diagnosticity of eyewitness confidence (Chan et al., 2022).

## Experiment 2

The goal of Experiment 2 was to address the potential concern that the source variability manipulation in Experiment 1 – via verbal instructions and a digit written on the cover page of the written transcript – was too weak to reveal an effect. To this end, we attempted to provide participants with more obvious source-specifying information in Experiment 2 by showing video interviews featuring different actresses.

### Participants

As in Experiment 1, we aimed to collect data from 44 participants per condition. A total of 367 participants completed the experiment, but data from 99 participants were removed based on the exclusion criteria listed in our preregistration (see Table 2). An additional 144 participants began the experiment but never completed it (~ 83% of these participants completed 0–1% of the experiment). The final sample contained 268 participants.

### Materials and procedure

**Figure 6:**
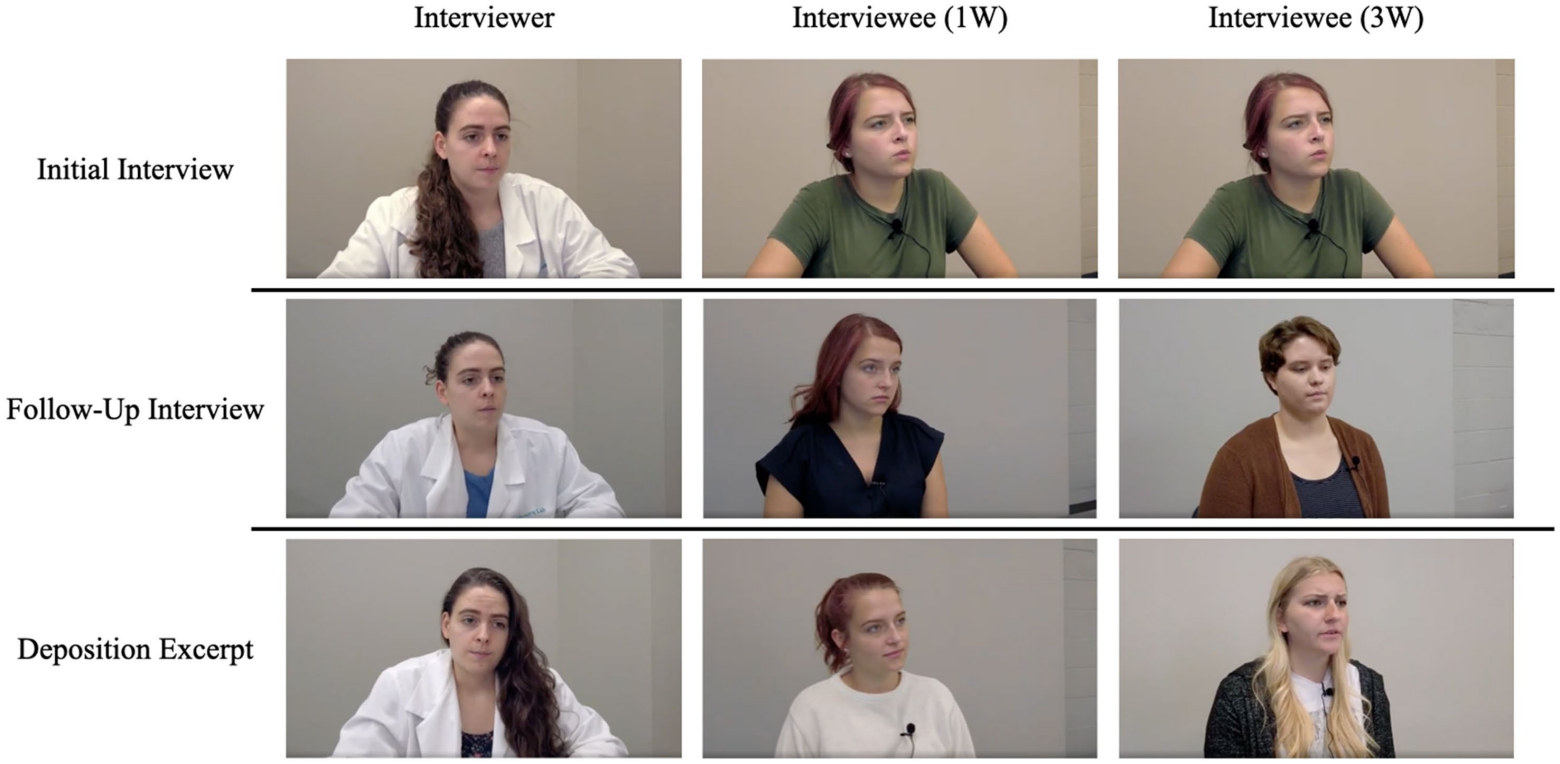
Depiction of interviewer and interviewees for 1W and 3W conditions by interview type. The interviewer remained the same across interviews. In the 1W condition, participants viewed the same witness wearing different clothes for each interview. In the 3W condition, participants viewed three different witnesses. The order of witnesses was randomized and counterbalanced.

The materials and procedure for Experiment 2 were identical to Experiment 1 except for the interview videos. Each video depicted an interviewer and an interviewee having a conversation. The scripts for the videos were identical to those in Experiment 1, with the addition of natural pauses and vocalized fillers (e.g., uhm, like, okay) to increase realism. Before each interview, participants were shown a statement that contained the witness identifier and the day on which the interview took place. At all times, the videos showed either the interviewee or the interviewer (see Figure 6). Three women acted as interviewees, and their order of appearance across the interviews was counterbalanced across participants. Because participants were told that the interviews occurred across three days, all actresses wore different clothes for each interview.

### Results and discussion

Accuracy for the filler questions is presented in Table 3. Because accuracy did not significantly differ in the 1X condition regardless of whether the misinformation was presented in the first or third interview (all *t*s < 1.04, *p*s > 0.304), all analyses reported below were collapsed across this variable.

#### The effect of repetition and source variability on accuracy

##### Replication of Foster et al.’s conditions

**Figure 7:**
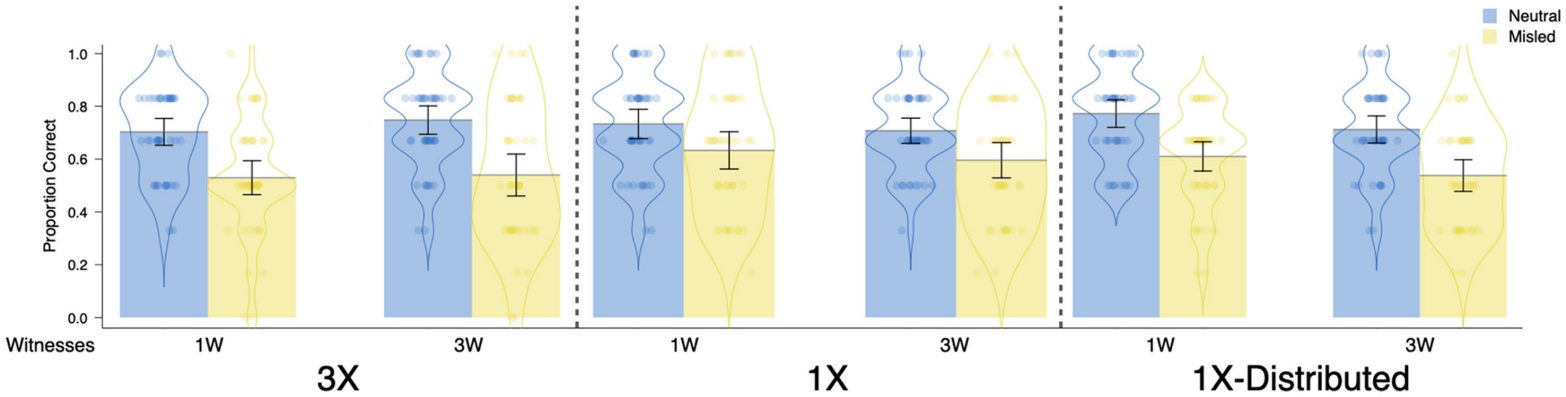
Proportion correct by item type in Experiment 2 as a function of repetition and sources. Each dot represents the data of an individual participant. Jitter was introduced to disperse the data points horizontally for visualization purposes. The violin element displays data density.

Figure 7 shows the critical findings from Experiment 2. Replicating Experiment 1, repetition of misinformation reduced participants’ accuracy. However, contrary to our expectations (given that we increased the salience of manipulation), we did not find an effect of source variability. That is, there was no difference in participants’ recognition accuracy regardless of whether they received misinformation from three people or from one person.

In other words, a repeated measures ANOVA (the same as in Experiment 1) demonstrated a main effect of item type, *F*(1, 176) = 54.39, *p* < 0.001, *d* = 0.55, which revealed a misinformation effect, such that participants were less accurate for misled items (*M* = 0.58, *SD* = 0.24) than for neutral items (*M* = 0.72, *SD* = 0.17). Moreover, there was an interaction between item type and repetition, *F*(1, 176) = 4.45, *p* = 0.036, η_p_^2^ = 0.03. All other main effects and interactions were not significant, *F*s < 2.57, *p*s > 0.111.

The interaction between item type and repetition demonstrated that we again replicated the critical results of Foster et al. (2012), such that repetition of misinformation reduced participant’s accuracy on the misled items (*M*_1X_ = 0.61, *M*_3X_ = 0.54), *t*(178) = 2.29, *p* = 0.023, *d* = 0.34, but not on the neutral items (*M*_1X_ = 0.72, *M*_3X_ = 0.73), *t*(178) = 0.20, *p* = 0.843, *d* = 0.03. In addition, although we expected an effect of source variability with the more powerful manipulation in this experiment, increasing the variability of sources did not lead to any significant differences in participants’ recognition accuracy for both misled (*M*_1W_ = 0.58, *M*_3W_ = 0.57), *t*(178) = 0.38, *p* = 0.702, *d* = 0.06, and neutral items (*M*_1W_ = 0.72, *M*_3W_ = 0.73), *t*(178) = 0.37, *p* = 0.712, *d* = 0.06. We further examined the implications of this null effect in the General Discussion.

##### Comparisons that controlled for the distribution of misinformation across interviews

The right side of Figure 7 shows the critical results for these conditions. Overall, contrary to our expectations, we did not find an effect of either the repetition or source variability manipulations. That is, participants’ recognition accuracy was not influenced by either repetition or source variability.

In other words, when we conducted the analysis to examine the effects of repetition and source variability on accuracy for the 3X conditions and the 1X distributed conditions (see the right side of Figure 7), the analysis only revealed a misinformation effect, *F*(1, 174) = 89.99, *p* < 0.001, *d* = 0.71, and a surprising interaction between repetition and source variability, *F*(1, 174) = 4.35, *p* = 0.039, η_p_^2^ = 0.02. We caution against overinterpreting this interaction given that (i) we did not predict it, and (ii) the effect was small, and (iii) this interaction collapsed across item type, which was the most influential variable. But perhaps most importantly, repetition and item type did not interact, *F*(1, 174) = 0.35, *p* = 0.553, η_p_^2^ = <0.01. All other main effects and interactions were nonsignificant, *F*s < 1.56, *p*s > 0.213.

Contrary to our expectations, we did not find that repetition of misinformation decreased recognition accuracy for the misled items (*M*_1X_ = 0.57, *M*_3X_ = 0.54)*, t*(176) = 1.21, *p* = 0.229, *d* = 0.18, and as expected, repetition of misinformation did not influence accuracy for the neutral items (*M*_1X_ = 0.74, *M*_3X_ = 0.73), *t*(176) = 0.65, *p* = 0.518, *d* = 0.10. In addition, we again found that the source variability manipulation did not influence recognition accuracy for both misled (*M*_1W_ = 0.57, *M*_3W_ = 0.54), *t*(176) = 0.94, *p* = 0.348. *d* = 0.14, and neutral items (*M*_1W_ = 0.74, *M*_3W_ = 0.73), *t*(176) = 0.27, *p* = 0.786, *d* = 0.04.

#### Confidence-accuracy calibration by item type

**Figure 8:**
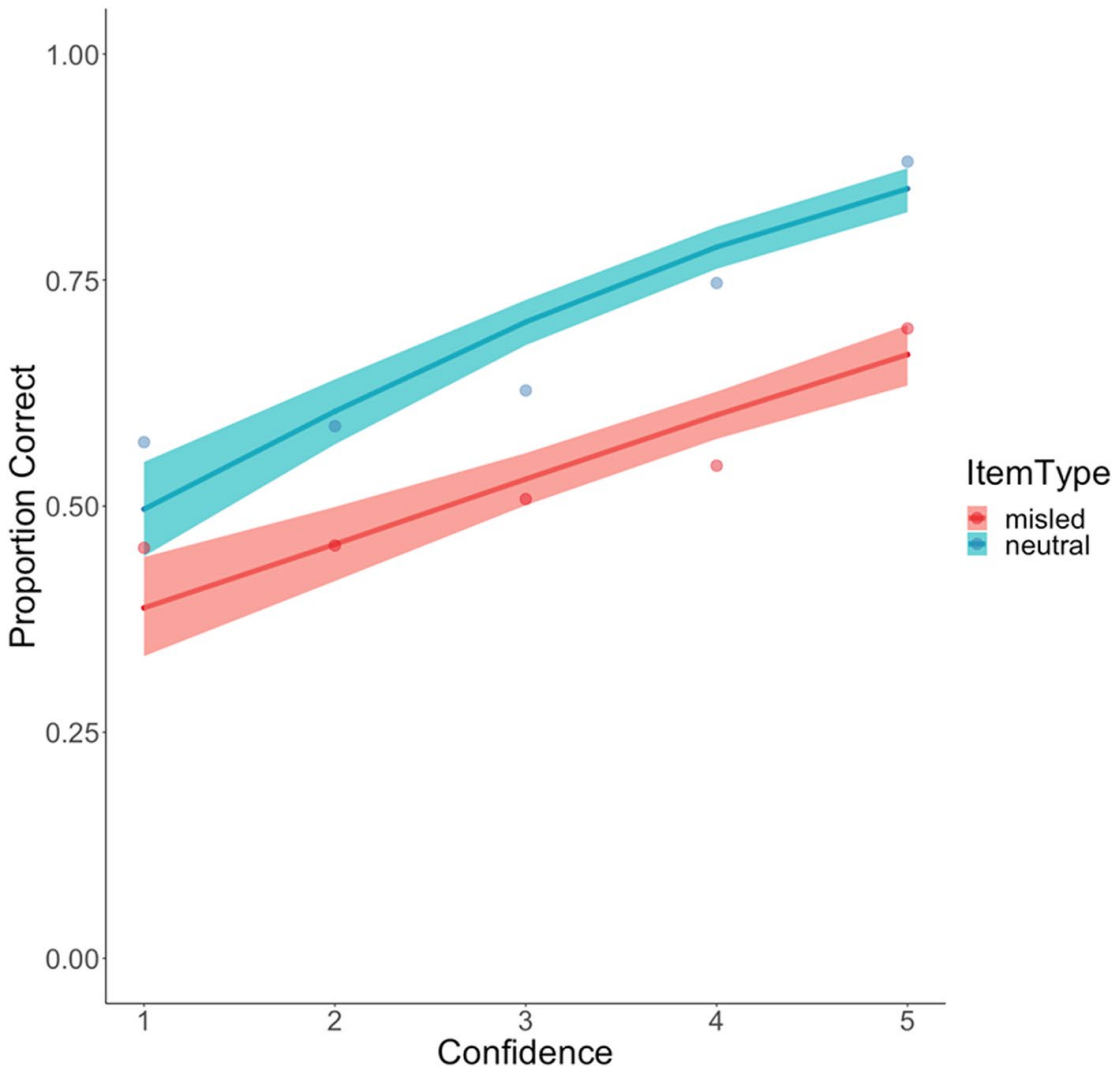
Confidence accuracy calibration as a function of item type in Experiment 2. Data points represent observed probabilities, and bands represent 0.95 CI for the fitted multilevel regression lines.

In Experiment 1, we found that misinformation flattened the confidence-accuracy relationship in participants’ responses. We conducted the same multilevel logistic regression for the data in Experiment 2 and found a similar pattern of results. Specifically, there is a significant interaction between confidence and item type, *B* = 0.15, *SE* = 0.06, *z* = 2.63, *p* = 0.008. The confidence-accuracy relationship was stronger for neutral items, *B* = 0.44, *SE* = 0.04, *z* = 10.67, *p* < 0.001, than for misled items, *B* = 0.29, *SE* = 0.04, *z* = 7.18, *p* < 0.001, although this difference was not as dramatic as that in Experiment 1 (see Figure 8). When we examined the observed data, it was clear that recognition performance remained close to chance for the *misled items* across confidence levels 1 to 4, but participants achieved substantially better performance when they reached the highest level of confidence. In contrast, for the *neutral items*, recognition accuracy was consistently above chance and rose with increasing confidence.

Overall, in Experiment 2, we replicated the pattern of results shown in Foster et al. (2012), namely, that repetition of misinformation harmed recognition accuracy. But in the 3X vs. 1X distributed comparison, we did not replicate this effect. It is unclear why participants in Experiment 2’s 1X distributed condition selected the misinformation at a higher rate than was typical compared to the other 1X conditions in this study. With no better explanation, we believe this result can be attributed to a sampling error. Moreover, despite increasing the salience of the source manipulation, presenting misinformation from one or three witnesses did not influence participants’ accuracy. Finally, we consistently demonstrated that the confidence-accuracy relationship was well-calibrated for neutral items, but the introduction of misinformation flattened this relationship.

### Does repeating misinformation increase its influence? A meta-analysis

Repetition effects have a long history in memory research (since Ebbinghaus, 1964, originally published in 1885) and have been studied quite extensively in the literature on erroneous memory. For example, research on imagination inflation (e.g., Garry et al., 1996; Thomas et al., 2003) and ironic effects of repetition (e.g., Jacoby, 1999; Benjamin, 2001) showed that repetition can drive false remembering. As we have reviewed in the Introduction, several studies have shown that repetition of misinformation can increase its influence. In the current study, three of our four comparisons revealed a significant repetition effect. Overall, we found that repetition increased the misinformation effect, although, as described previously, one comparison in Experiment 2 produced a null effect. To further contextualize the repetition effect in the misinformation literature, we examined extant studies that have used repetition manipulations to provide a meta-analytic estimate of the effect size.

The literature of repetition effects on memory is enormous. It is therefore important to define and constrain the criteria for inclusion to make this meta-analysis feasible. To be faithful to the *misinformation effect design*, we included only studies in which the *to-be-rejected* (i.e., misleading) materials were repeated after a neutral encoding phase (that did not include misinformation). In addition, studies were excluded if the encoding phase was interactive – that is, if a participant engaged with the experimenter during the back-and-forth phases. These studies mainly included children as the participants, presumably to keep participants engaged during the encoding event. Together, our selection criteria excluded DRM or inference-driven false memory studies (Benjamin, 2001; McDermott and Chan, 2006), in which the to-be-rejected items were never presented for encoding. We also excluded studies that repeated the to-be-remembered (rather than to-be-rejected) items during encoding, such as studies that demonstrated the illusory truth effect (Arkes et al., 1991; Hassan and Barber, 2021), repetition effects in verbal learning (Howe, 1970, 1972; Melton, 1970; Jacoby, 1978; Hintzman, 2010), studies that demonstrated the imagination inflation effect (Garry et al., 1996; Goff and Roediger, 1998; Thomas et al., 2003), or studies that use a repeated retrieval (rather than repeated encoding) procedure to induce false memories (Poole and White, 1991; Shaw et al., 1995; Memon and Vartoukian, 1996; Roediger et al., 1996; Hauer et al., 2007; Henkel, 2007, 2008; Hershkowitz and Terner, 2007; La Rooy et al., 2010; Chan and Langley, 2011).

To find articles, we searched PsycINFO, PsycArticles, PubMed, and Google Scholar with the following search terms: “misinformation effect AND repetition,” “repeated (or repetition of) misinformation,” and “misinformation AND repetition.” We also searched for studies that either cited or were cited by Mitchell and Zaragoza (1996) or Foster et al. (2012). Among the search results, we included only studies that used the misinformation effect procedure and involved a repetition manipulation (typically once vs. two or three presentations). Finally, we included only studies that contained enough information to calculate effect sizes (accuracy under one presentation of misinformation vs. accuracy under multiple presentations of misinformation).

In total, 19 effect sizes from 12 studies were included in this meta-analysis. Data from the current experiments were collapsed across the 1X variable for both Experiments 1 and 2 to avoid over-representing data from the 3X group.^4^ Studies included in the meta-analysis are marked with an asterisk in the Reference section. Most included studies either directly reported an effect size of repetition or reported enough information for an effect size to be derived. For one study (Mitchell and Zaragoza, 1996), standard deviation was not reported (resulting in not enough information to calculate an effect size), so we imputed their standard deviation based on the remaining studies in the meta-analysis. We used the “meta” package in R to conduct the meta-analysis. As we anticipated heterogeneity between studies, we used a random-effects model to pool effect sizes. In addition, we used the restricted maximum likelihood estimator to calculate the heterogeneity variance τ^2^ and the Knapp-Hartung adjustments to calculate confidence interval around the pooled effect.

**Figure 9:**
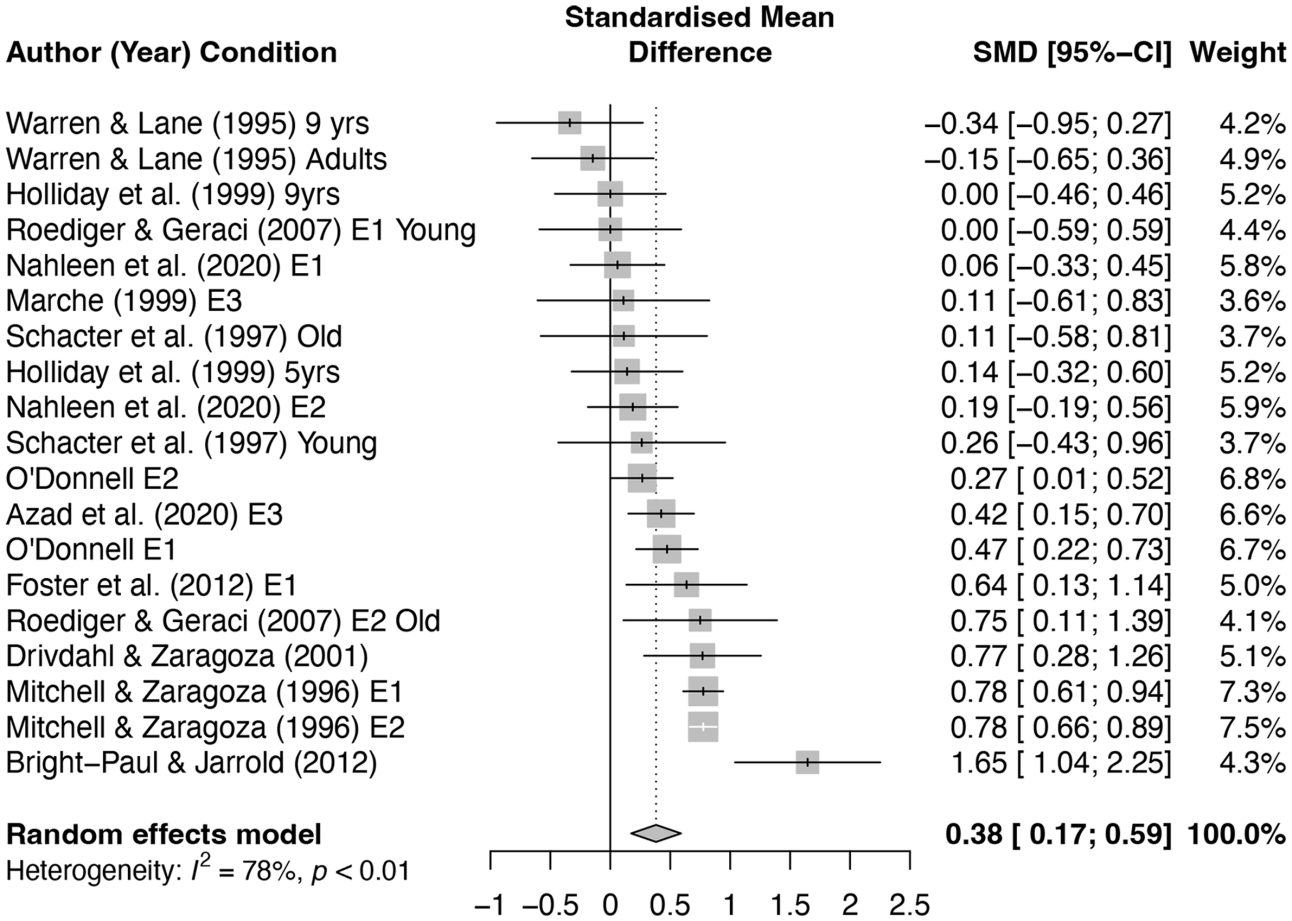
A forest plot of repetition effect sizes for misinformation effect studies.

Figure 9 shows a forest plot of this meta-analysis, with the random effects model producing a moderate repetition effect, *g* = 0.38 [0.18, 0.59], *p* < 0.001. A majority of the sampled studies showed a positive repetition effect,^5^ and only two effect sizes were negative. We conducted an Egger’s test, *t*(17) = −2.98, *p* = 0.01, (in R using the metabias function) to examine asymmetry and found some evidence of asymmetry in the funnel plot, although this did not necessarily indicate publication bias. That is, given the negative result from the above analysis, it is clear from examining the funnel plot (see Supplementary material, Figure 1) that the data trended in the opposite direction of publication bias, as demonstrated by the concentration of data in the bottom left of the plot. Note that this meta-analysis was not meant to be exhaustive but was designed to provide a broader, quantitative examination of the extant effect sizes regarding repetition and the misinformation effect. A note about the existing literature: perhaps due to practical constraints, most studies had compared a single presentation of misinformation to either two or three presentations, so we currently do not know if the repetition effect is monotonically positive, if it would reach an asymptote, or if it would take on an inverted U function, such that repeating the same misinformation “too many” times would reduce its influence, similar to how being exposed to “too many” pieces of misinformation can increase people’s resistance to the misinformation (Pena et al., 2017). Further research is needed to address this important question.

### A meta-analysis of source variability

In the current study, we failed to observe a source variability effect. The null effect in Experiment 2 was particularly notable because we expected that making clear that three different people (instead of a single person) provided the same misinformation would strengthen its influence. One argument that could be raised is that the null effect of Experiment 2, despite our best effort to make the witnesses clearly different, could still have been the result of a weak source manipulation. That is, one might argue that we could further increase the distinctiveness of the sources by further distinguishing the witnesses in terms of gender, age, and other obvious characteristics. Although each of the confederates was female and around the same age (18–22), it is very unlikely that they were indistinguishable. Each confederate had a different hair color (red, blond, and brown) and style, all three used different mannerisms, and each interviewee had a different voice. Thus, it is unlikely that the null effect of source variability is attributable to a weak source manipulation.

**Figure 10:**
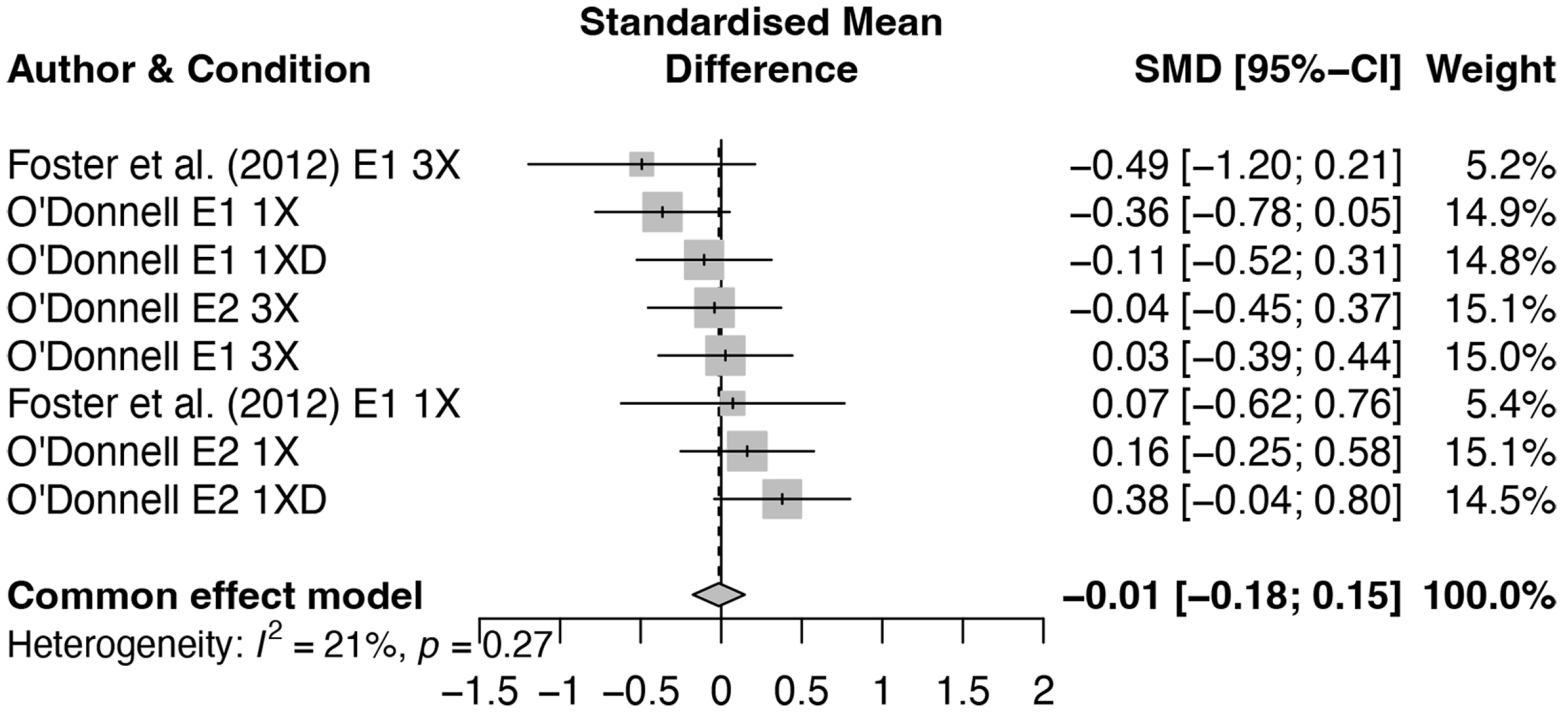
A forest plot of the source variability effect in the current study and Foster et al. (2012) by repetition condition.

We believe that a more likely argument is that source variability by itself does not significantly influence accuracy or suggestibility. Across four experimental comparisons involving misled items, we did not find any significant effects of the source manipulation. Notably, these null effects occurred regardless of whether each piece of misinformation was presented only once (which allowed us to examine the influence of source variability on its own) or three times (which allowed us to examine the effect of source variability in the context of repeated misinformation). In addition, when we conducted a meta-analysis using the data from both the current study and Foster et al. (2012), a fixed effects model showed that the *source variability effect* was essentially nil, *g* = −0.01 [−0.18, 0.15], *p* = 0.856 (See Figure 10). For this meta-analysis, we did not conduct an Egger’s test, given that there were not enough studies to conduct this test and that there was only one published study included. However, the funnel plot (see Supplementary material, Figure 2) does not show evidence of asymmetry. We used the same package in R to conduct this analysis but used a fixed-effects model, given that both studies used largely the same design. In addition, we applied *ad hoc* variance correction, given the small Hartung-Knapp variance estimate. Furthermore, the current experiments demonstrated the same null effect shown in Foster et al. (2012) with greater statistical power. Altogether, the data from the current study and Foster et al. suggest that source variability had little to no discernible influence on participants’ memory, at least in conditions similar to those tested here.

## General discussion

In two experiments, we examined whether multiple presentations of the same misinformation and increasing the number of people who provide the same misinformation would affect people’s suggestibility. Overall, both experiments provided results that replicated Foster et al., such that repetition, but not source variability, increased the misinformation effect. One exception to this pattern was that repetition did not significantly harm performance when comparing the 3X against the 1X distributed condition in Experiment 2, which we attributed to a sampling error. Moreover, across both experiments, participants exhibited a strong, positive confidence-accuracy relationship for the neutral items, but exposure to misinformation severely depressed this relationship, such that only the most confident responses exceeded chance level accuracy.

In the remainder of the General Discussion, we briefly review the results of our repetition manipulation, then discuss the potential reasons why there were no differences in accuracy when participants read or watched interviews from three witnesses compared to one witness, and finally, consider the applied implications of these findings.

### The effect of repeated misinformation on suggestibility

In Experiment 1, participants read three interview transcripts that either introduced them to misinformation once or three times. In Experiment 2, the interview transcripts were formatted as videos, and participants were again introduced to misinformation either once or three times. Across both experiments, the effect of repetition was reliable but modest (*g* = 0.47 in Experiment 1 and *g* = 0.27 in Experiment 2). Indeed, the repetition effect was only significant in one of two comparisons in Experiment 2. It should be noted that the current studies were powered to detect a larger effect size than we found. However, the meta-analysis demonstrated a significant, moderate effect of repetition among studies in the misinformation literature that used a similar paradigm. Moreover, although the current studies demonstrated a smaller effect size than that of Foster et al. (*g* = 0.64), a sampling of the literature shows a variety of effect sizes, and indeed, our effect sizes fell squarely inside the 0.95 CI of the meta-analytic effect of 0.18 to 0.59.

A practical note is that our manipulation of repetition, and indeed the repetition manipulation implemented in most misinformation studies to date, is relatively weak. We had participants read/watch someone reproduce the same piece of misinformation up to three times within a 10–20 min span. In actual criminal investigations, an eyewitness might be exposed to the same piece of misinformation on far more occasions stretched across a much longer interval (Barry et al., 2017). Given that spaced presentations enhance learning relative to massed presentations (Cepeda et al., 2006), real-life eyewitnesses who are repeatedly exposed to the same piece of misinformation across a longer time interval than is typical in laboratory settings might demonstrate a greater repetition effect. Future research should examine the influence of varying number of repetitions and the intervals between repetition of misinformation on eyewitness suggestibility.

### Does varying misinformation sources increase its influence?

In the present experiments, − and in Foster et al. (2012) – increasing the number of misinformation sources did not affect participants’ suggestibility. In addition, a meta-analysis of the data from both the current studies and Foster et al. demonstrated that, among eight effect sizes, the effect of source variability was essentially nil. Of course, source variability might yet produce an effect in future studies with heretofore unexamined variables (such as with even more eyewitnesses who deliver the misinformation, with a source memory test, with different participant populations, or if participants discussed the details of the encoding event with confederates).

Assuming that source variability does not normally affect eyewitness memory, what might explain this null effect? Foster et al. (2012) theorized that people might not account for the number of eyewitnesses who make a statement during memory retrieval. Rather, they rely on the fluency or familiarity of the information they are retrieving without recalling where the information came from or how many sources contributed to the information. This idea is supported by other research (Thomas et al., 2010; Chan et al., 2012), including studies that showed that perception of consensus is driven primarily by the fluency of the retrieved information (Weaver et al., 2007; Schwarz et al., 2021). By this logic, information that is given once by three different sources would produce a similar feeling of consensus as information that is repeated three times by the same source. Finally, across six experiments, Weaver et al. demonstrated that repeatedly presenting one piece of information gave participants a sense of consensus for that information, even though only one person presented it. In these experiments, participants read focus group opinion statements. In Experiments 1 and 5, participants either read one opinion from one person, a similar opinion three times from the same person, or a similar opinion from three different people (participants were told these opinions were sampled from a focus group of five people). Experiments 2a, 2b, 3, and 4 contained only the first two conditions listed above. After reading the statements, participants were asked to estimate the opinions of the focus group on a 1–7 Likert scale. Participants consistently perceived an opinion to have more support when it was repeated by the same person, despite knowing that only one person was providing the opinion (as each opinion had a name attached to it). This result suggests that participants relied on fluency when making consensus judgments. As such, it could be that the null effect of source variability was a result of participants ascribing similar feelings of consensus to information that was presented three times by one person and information that was presented once each by three different people.

Additionally, Foster et al. (2012) argued that different mechanisms might contribute to the credibility of information from one source compared to information from three sources. Specifically, when one witness repeats a claim, the person may be judged as more credible (than a person who does not repeat a claim) because of the consistency exhibited *across instances* (Chan et al., 2017; Smelter and Calvillo, 2020). When three witnesses make the same claim, it may be judged as credible because of the consistency exhibited *across individuals* (Lindsay et al., 1986). Thus, a claim that has been repeated by a single witness may be perceived as more accurate because the *witness* is deemed consistent, whereas perceptions of accuracy in a claim that is made by three different witnesses may be judged as more accurate because *the claim itself* is viewed as more credible than a claim that has not been uttered by multiple witnesses. Ultimately, however, it is the repetition, not variation of sources, that increases the credibility of a claim.

The lack of a source variability effect suggests that participants might not have used source-specifying information during their retrieval, but it is also possible that this null effect occurred because participants were not given explicit instructions to use source specifying information. A notable difference between the results of Foster et al.’s (2012) and Mitchell and Zaragoza (1996), aside from the differences in the materials and the nature of the source manipulation,^6^ was that Mitchell and Zaragoza gave participants a source discrimination test, whereas Foster et al. and (the current experiments) gave participants a 2AFC recognition test. Therefore, it is possible that a source variability effect might only surface when participants were forced to consider source-specifying information, such as when they were told explicitly to retrieve contextual information or when they were provided with explicit warnings. Future research can address this possibility by manipulating both source variability and retrieval requirements.

### Applied implications

From an applied perspective, our results indicate that an individual who hears a piece of misinformation repeatedly would be more likely to report that misinformation than an individual who hears the same piece of misinformation only once, and this is true regardless of how many sources repeatedly present the misinformation. More broadly, an intriguing implication of these findings is that attempts to retract or debunk misinformation should avoid including the misinformation (e.g., correcting the misinformation without explicitly restating it), given that repetitions can increase suggestibility. Research on the continued influence effect has examined this possibility with somewhat mixed results. Several researchers have found that including the details of the misinformation in a retraction can have a backfire effect, such that people often falsely remember the information being corrected as true (Skurnik et al., 2005; Nyhan and Reifler, 2010; Peter and Koch, 2016). However, more recent research showed that retractions that included the misinformation were more effective at reducing the continued influence effect than retractions that did not mention the misinformation (Ecker et al., 2017, 2020). A possible explanation for these discrepant findings is that timing of the correction matters. In studies that did not demonstrate a backfire effect, participants read statements or new articles and received a correction (with or without a reminder of the fake news) *after a delay* (Ecker et al., 2017, 2020). In contrast, in the studies that demonstrated a backfire effect, participants usually read statements or news articles with a truth verification *simultaneously*.

The current experiments showed that participants were generally quite adept at judging their response accuracy, as they demonstrated a positive confidence-accuracy relationship for the neutral items (see Figures 5, 8), which is consistent with recent findings in the literature (Wixted et al., 2018). But perhaps more remarkable was the much flatter confidence-accuracy curves for the misled items. In line with Wixted et al.’s argument, the confidence-accuracy relationship was flatter when it was contaminated by misinformation. In both experiments, participants exhibited near-chance performance for these items, and accuracy only exceeded chance at the highest level of confidence. This poor confidence-accuracy relationship shows the evidence-contaminating power of misleading suggestions and replicates recent findings that showed that, in the absence of a warning, misinformation can damage *both the accuracy* of eyewitness memory reports *and the diagnosticity* of eyewitness confidence (Chan et al., 2022).

## Conclusion

In two preregistered, high-powered experiments, we attempted to conceptually replicate Experiment 1 of Foster et al. (2012) and determine whether both repetition and source variability influence eyewitness suggestibility. In three of the four comparisons, we demonstrated a significant effect of repetition,^7^ and our small-scale meta-analyses provided further evidence that repetition of misinformation can exacerbate eyewitness suggestibility.

In contrast to the effect of repetition, we found no effect of source variability. Although one might suggest that this null effect was the result of a weak manipulation, we argue here that our manipulation produced obvious differences across the three interviewees in an ecologically realistic manner. We therefore conclude that, as Foster et al. (2012) did, it is repetition of the misinformation that increases an eyewitness’s suggestibility, not the number of people who provide the misinformation.

## Data availability statement

The datasets presented in this study can be found in online repositories. The names of the repository/repositories and accession number(s) can be found at: https://osf.io/9zpfk/?view_only=f95ed70720c742d48296fa3b92891ed7.

## Ethics statement

The studies involving human participants were reviewed and approved by the Institutional Review Board at Iowa State University. The patients/participants provided their written informed consent to participate in this study. Written informed consent was obtained from the individual(s) for the publication of any potentially identifiable images or data included in this article.

## Author contributions

RO’D and JC conceived the research idea. RO’D adapted the master’s thesis (O’Donnell, 2022), which was written to fulfill the requirements of her master’s degree during her Doctoral Program in Psychology at Iowa State University, collected and analyzed the data for both experiments under the guidance of JC, and wrote the full first draft of the manuscript. JC provided revisions. MG and JF provided consultations on the project, suggestions regarding the design and procedure, and comments on the manuscript. All authors contributed to the article and approved the submitted version.

## Conflict of interest

The authors declare that the research was conducted in the absence of any commercial or financial relationships that could be construed as a potential conflict of interest.

## Publisher’s note

All claims expressed in this article are solely those of the authors and do not necessarily represent those of their affiliated organizations, or those of the publisher, the editors and the reviewers. Any product that may be evaluated in this article, or claim that may be made by its manufacturer, is not guaranteed or endorsed by the publisher.

## Footnotes

^1^Instead of eyewitness memory, Experiment 2 in Foster et al. concerned jury decision making, so it is not relevant to our research purpose.

^2^An additional 112 participants began the experiment but never completed it (91% of these participants completed only 1–2% of the experiment).

^3^Participants reported how many witnesses they believed had been interviewed and rated the credibility, accuracy, and consistency of each witness on a 1–5 Likert scale. Unfortunately, in hindsight, the questions were poorly phrased. Participants clearly did not interpret the questions correctly, as indicated by their answers to the question “How many eyewitnesses were interviewed?” (*M* = 8.39, *SD* = 5.89, range = 0–32).

^4^We chose this method to prevent the data from the 3X condition from being included twice. If we had instead treated the data from each condition separately, we would have computed an effect for the 1X vs. 3X comparison and another effect size for the 1X distributed vs. 3X comparison.

^5^One might wonder whether Bright-Paul and Jarrold (2012) is an outlier. An analysis conducted without that study resulted in a slightly smaller, but still significant effect size of *g* = 0.34 [0.17, 0.51], *p* < 0.001.

^6^Note again, however, Mitchell and Zaragoza did not manipulate the number of witnesses who provided the same piece of misinformation; rather, they manipulated presentation modality.

^7^Note that in all four comparisons, repetition reduced recognition accuracy for the misled items quantitatively.
