# Supplementary material for: Experimental and meta-analytic evidence that source variability of misinformation does not increase eyewitness suggestibility independently of repetition of misinformation
Source: Front Psychol. 2023 Aug 24;14:1201674. doi: 10.3389/fpsyg.2023.1201674 (PMC10492197; doi:10.3389/fpsyg.2023.1201674)
Supplement: Supplementary file 1 [file Image_1.PDF]

1.1 Supplementary Figures

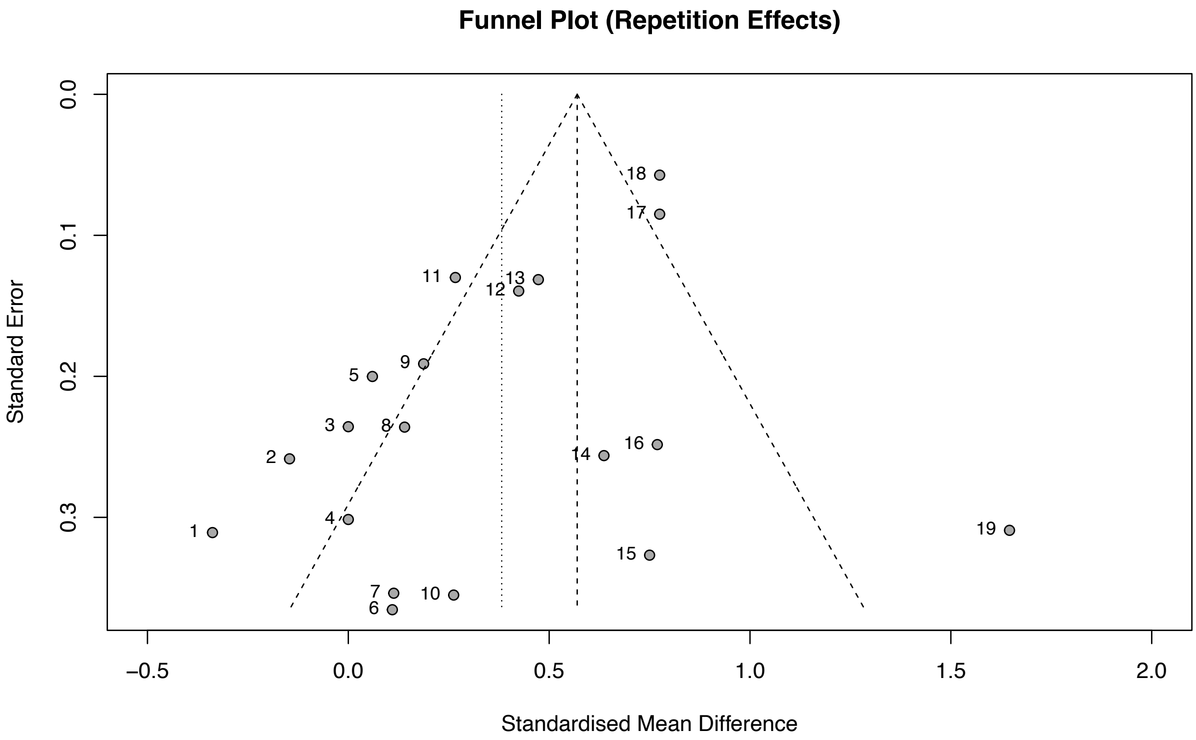

Supplementary Figure 1. Funnel plot for the repetition effects meta-analysis. The funnel plot is labeled with the numbers that appear under the FunnelValue column in the Repetition Meta-Analysis Excel file on OSF. Experiments are numbered from the smallest effect size to the largest effect size.

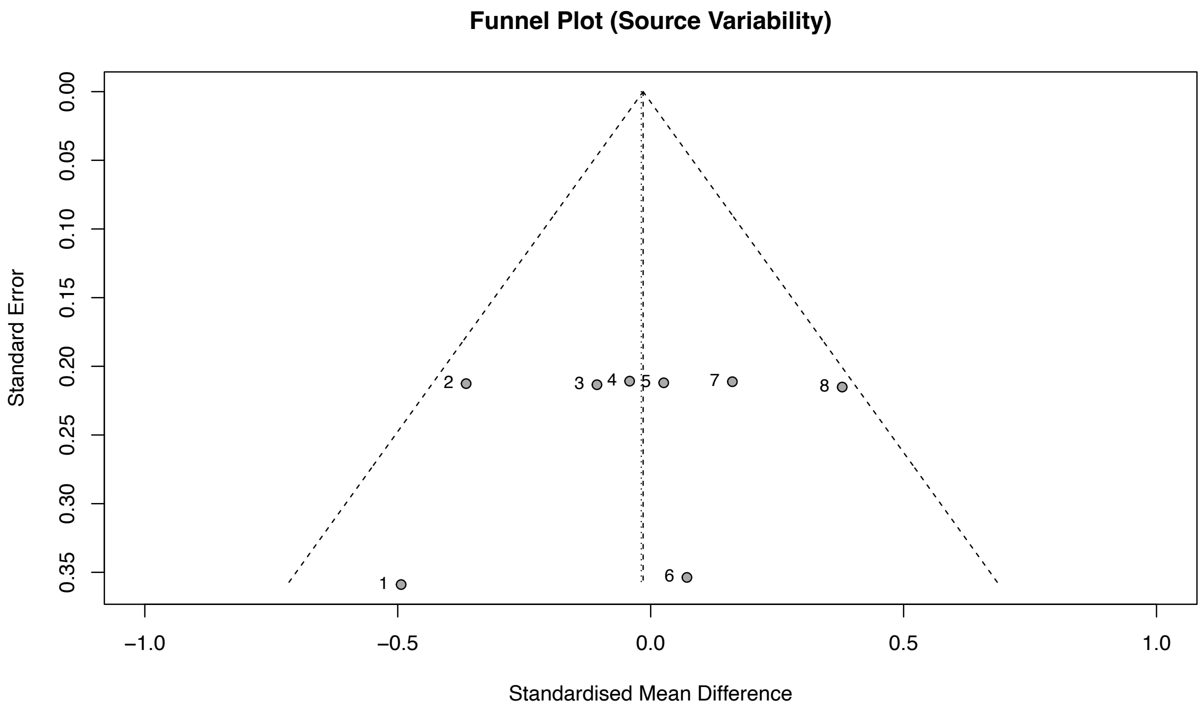

Supplementary Figure 2. Funnel plot for the source variability meta-analysis. The funnel plot is labeled with the numbers that appear under the FunnelValue column in the Source Variability Meta-Analysis Excel file on OSF. Experiments are numbered from the smallest effect size to the largest effect size.
